# Effect of Selenium on Lipid and Amino Acid Metabolism in Yeast Cells

**DOI:** 10.1007/s12011-018-1342-x

**Published:** 2018-04-19

**Authors:** Marek Kieliszek, Stanisław Błażejak, Anna Bzducha-Wróbel, Anna M. Kot

**Affiliations:** Faculty of Food Sciences, Department of Biotechnology, Microbiology and Food Evaluation, Warsaw University of Life Sciences − SGGW, Nowoursynowska 159 C, 02-776 Warsaw, Poland

## Abstract

This article discusses the effect of selenium in aqueous solutions on aspects of lipid and amino acid metabolism in the cell biomass of *Saccharomyces cerevisiae* MYA-2200 and *Candida utilis* ATCC 9950 yeasts. The yeast biomass was obtained by using waste products (potato wastewater and glycerol). Selenium, at a dose of 20 mg/L of aqueous solution, affected the differentiation of cellular morphology. Yeast enriched with selenium was characterized by a large functional diversity in terms of protein and amino acid content. The protein content in the biomass of *S. cerevisiae* enriched with selenium (42.6%) decreased slightly as compared to that in the control sample without additional selenium supplementation (48.4%). Moreover, yeasts of both strains enriched with selenium contained a large amount of glutamic acid, aspartic acid, lysine, and leucine. Analysis of fatty acid profiles in *S*. *cerevisiae* yeast supplemented with selenium showed an increase in the unsaturated fatty acid content (e.g., C18:1). The presence of margaric acid (C17:0) and hexadecanoic acid (C17:1) was found in the *C*. *utilis* biomass enriched with selenium, in contrast to that of *S*. *cerevisiae*. These results indicate that selenium may induce lipid peroxidation, which consequently affects the loss of integrity of the cytoplasmic membrane. Yeast enriched with selenium with optimal amino acid and lipid composition can be used to prepare a novel formula of dietary supplements, which can be applied directly to various diets for both humans and animals.

## Introduction

Selenium is an essential element in the physiological functions of humans and animals [1]. However, excessive consumption of selenium may cause disturbances in mechanisms of homeostasis and could even be fatal. The bioavailability of selenium in living organisms and its beneficial effects depend, to a large extent, on the chemical form and its quantity in food [2–4]. Selenium is characterized by a narrow therapeutic index [5]. The right dose of this element positively affects human health and prevents the risk of cancer, cardiovascular diseases, diabetes, and infertility [6, 7]. Selenium supports cell-cycle progression and is essential for optimal immune response [8].

Depending on culture conditions, yeast cells can bioaccumulate selenium in various cell structures, and then convert it into organic forms [2]. Selenium in food is usually observed in the form of amino acids: selenocysteine and selenomethionine (SeMet). The organic forms of selenium are more easily absorbed by organisms as compared to inorganic forms [8]. Appropriate levels of selenium in humans and animals protect against lipid peroxidation, accumulation of compounds containing carbonyl groups, and protein fragmentation caused by oxidative stress [5, 9, 10].

A growing interest in dietary supplements and the dynamic development of the market for nutraceuticals enriched with microelements is being observed nowadays. Some of these additives contain inorganic salts of selenium, mainly as sodium selenite (Na_2_SeO_4_) and sodium selenite (Na_2_SeO_3_) forms, whereas other preparations are based on yeast enriched with organic selenium. The production of yeast biomass from the waste products of various industries as components of the culture medium yields a cost-effective and economically advantageous process. Yeast cultivation from waste substrates, such as waste from the agro-food industry (potato wastewater) that can be transformed into more valuable products, is becoming an increasingly popular technology. Yeast can convert more than 80% of inorganic selenium into its organic form, such as SeMet [3, 11]. Selenium—in combination with proteins—forms the so-called selenium bioplexes, which have better intracellular absorbability and incorporability [12]. For these reasons, yeast biomass is an economical and easily available source of organic forms of selenium [5].

Under specific culture conditions, many microorganisms demonstrate the ability to produce fat via glycerol esterification. Yeast can accumulate neutral lipids, which are stored in the form of liposomes (lipid droplets) [13]. Of note, the composition of lipids depends on both culture conditions and composition of the culture medium [14]. Thus, glycerol—exhibiting a lipophilic structure—is transported through cytoplasmic membranes of yeast cells by simple diffusion and then, via metabolic reactions, it can be transformed into valuable products such as unsaturated fatty acids [15].

This biosynthesis of polyunsaturated fatty acids by microorganisms has the potential to evolve into a novel, alternative source of these compounds. The combination of a yeast biomass enriched with essential microelements (selenium) and polyunsaturated fatty acids makes it possible to create new dietary supplements that can be used as additives in human nutrition or, in the form of a cellular biomass, as a high-energy feed additive for animals. Moreover, the intensive development of a new field of knowledge called lipid (liposomal) technology involves intraliposomal encapsulation of proteins. Lipid droplets are capable of enclosing biologically active substances (selenium proteins) and then participating in their controlled delivery to various tissues of the body. In recent years, a few papers have discussed this topic in the literature. Research into this aspect involves the possibility of unearthing an effect of selenium on the lipid profile in yeast cells. This is an interesting research area that can be the basis for developing alternative and innovative technologies for yeast biomass production. Thus, yeast can be treated as a valuable source of selenium bioplexes and lipids with a favorable fatty acid profile.

This study was undertaken with an aim to evaluate the effect of selenium on the modification of fatty acids, proteins, and amino acid profiles in the biomass of *Candida utilis* (ATCC 9950) and *Saccharomyces cerevisiae* (ATCC MYA-2200) yeast derived with glycerol and potato wastewater as constituents of the culture medium.

## Materials and Methods

### Biological Material

This study used *S*. *cerevisiae* MYA-2200 and *C*. *utilis* ATCC 9950 yeast strains from the collection of pure cultures of the Department of Biotechnology and Food Microbiology, Warsaw University of Life Sciences. Biological material was stored on yeast extract peptone dextrose (YPD) slants at a temperature range of 6–8 °C.

### Microbiological Media

Potato wastewater (Food Processing Plant PEPEES S.A., Łomża) and glycerol (POCH, Poland) at a concentration of 5% by volume as the main source of carbon were used to prepare the culture medium. An aqueous solution of Na_2_SeO_3_ salt (1000 mg Se^4+^/L) was added to aqueous solutions in such volumes that the final concentration of selenium in 100 mL of solution was 20 mg/L. The deionized water filtered by the Milli Q system (Millipore, France) was used to prepare selenium solutions. The active acidity of media and aqueous selenium solutions was set at 5.0. All media were sterilized at 121 °C for 20 min.

## Yeast Cultures

### Preparation of Inoculation and Proliferation Cultures

Inoculation media were prepared through 24-h culture of *C*. *utilis* and *S*. *cerevisiae* yeast strains, taken with a loop from the YPD slant, on liquid medium containing potato wastewater and 5% glycerol. Cultures were incubated on a reciprocating shaker (SM-30 Control E. Büchler, Germany) at a vibration amplitude of 200 cycles/min at 28 °C for 24 h.

Then, 10% (*v*/*v*) of the cell suspension grown in an inoculation culture (5.0–8.0 × 10^8^ cfu/mL) was used for inoculation of proliferation media (potato wastewater supplemented with glycerol). Culture conditions were the same as those for inoculation cultures for 24 h.

### Obtaining Yeast Biomass

The cell biomass of both yeast strains was obtained by centrifugation (3000×*g*, 10 min, 4 °C; Centrifuge 5804R, Eppendorf, Germany) of the propagation culture in a medium with glycerol and potato wastewater. The resulting yeast biomass was rinsed twice with sterile distilled water and centrifuged again.

We inoculated 10 g wet yeast biomass into 100 mL aqueous solutions enriched with selenium (20 mg Se^4+^/L). These cultures were grown under the same conditions as the inoculation and propagation cultures. After the 24-h culture process, the yeast biomass was centrifuged (3000×*g*, 10 min, 4 °C; Centrifuge 5804R, Eppendorf, Germany) and used in further analyzes.

### Determination of Protein Content in Yeast Biomass

The analysis of the total protein content in the yeast cell biomass was performed using the Kjeldahl method. The method involves mineralization of the sample, distillation, and titration of the ammonia that is released.

### Determination of Amino Acid Content in Yeast Biomass

The amino acid content in the yeast cell biomass was determined using an automatic amino acid analyzer (AAA 400 Ingos, Czech Republic, Prague) together with ion-exchange chromatography (Ostion Lg ANB columns). Ninhydrin in a citrate buffer was used in the post-column amino acid detection reactions. All samples were subject to acidic hydrolysis with 6 M HCl at 110 °C for 24 h. Samples for the determination of sulfuric amino acids were hydrolyzed separately with 6 M HCl after oxidation (formic acid and hydrogen peroxide, 9:1 by volume, for 16 h at 4 °C). After hydrolysis, the acid was evaporated in a vacuum evaporator under reduced pressure at 80 °C. The free HCl residue was dissolved in 2-mL loading buffer (0.2 M, pH 2.2) and injected into the amino acid analyzer. Individual analytes were derivatized in the reactor into colored amine–ninhydrin complexes. The identification was performed by a spectrophotometric detector at wavelengths of 570 and 440 nm by adjusting the retention times of the real samples and chromatographic standards. The quantitative analysis of amino acids was determined by comparing the area of the analyte peak with the surface (or height) of the standard peak as well as based on the calibration curves previously prepared using chromatographic standards (Sigma-Aldrich). Free amino acids determination was confirmed by comparing the retention times and peak areas of individual amino acids.

### Yeast Morphology Under a Transmission Electron Microscope

The centrifuged yeast cell biomass (3000×*g*, 10 min, 4 °C), obtained from aqueous solutions sans added selenium and then enriched with this element, was fixed in 2.5% glutaraldehyde for 2 h at 4 °C. The whole sample was rinsed in 0.025 M phosphate buffer at pH 7.2 for 2 h at 4 °C. The final stage of the fixing process was undertaken in 1% osmium tetraoxide solution for 1 h at 4 °C. The yeast cell biomass was dewatered in a rising gradient of ethyl alcohol and then supersaturated with acetone. After dehydration, the yeast biomass was embedded in epoxide EPON 812, and left for 24 h at room temperature. The material was then incubated at 60 °C for 48 h. Blocks with embedded material were cut with a diamond knife into ultrathin sections using an ultramicroton (LBK, Sweden). The ultrathin sections obtained were placed on microscopic (copper) grids, which were then contrasted with 9% uranyl acetate and 0.5% lead citrate. The specimens thus prepared were observed in a transmission electron microscope (JEM 1220 TEM; JEOL, Japan).

### Statistical Analysis

Results were subjected to an analysis of variance using Statgraphics Centurion XVII program. The significance of differences between mean values in particular groups was verified by Tukey’s HSD test for a significance level of *α* = 0.05.

## Results and Discussion

### Yeast Cell Morphology

**Fig. 1:**
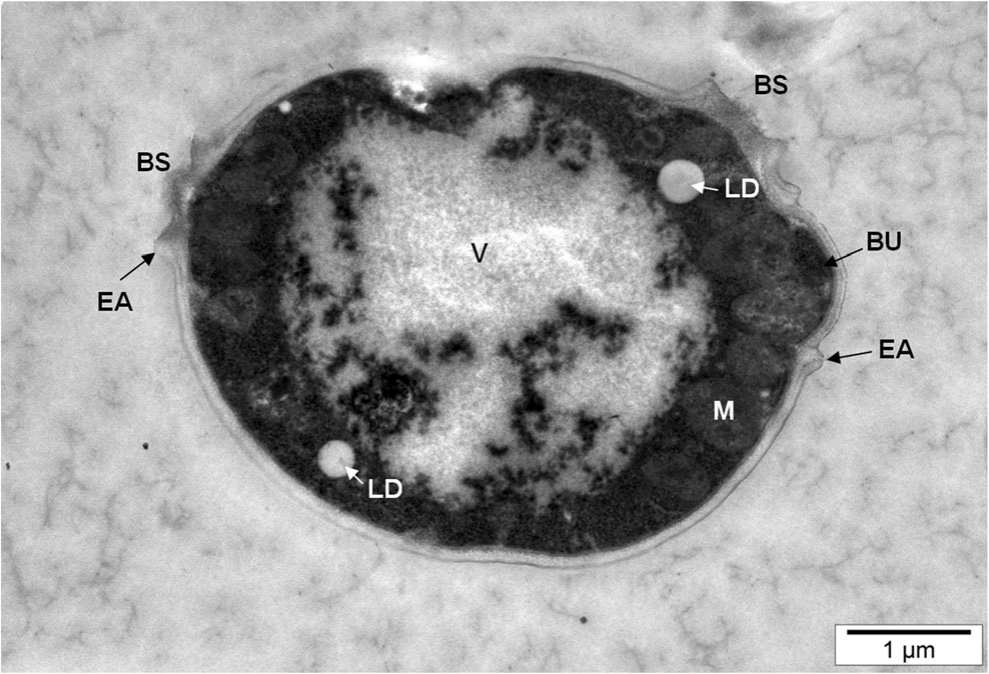
Image of *S*. *cerevisiae* yeast cells from 24-h culture in aqueous solutions (scars after budding)

A transmission electron microscope was used to assess the effect of selenium on the cell morphology of the yeast strains that were examined. A large diversity of cell morphological characteristics was observed. Mature yeast cells from the control medium evinced an ellipsoid shape with thick rounded edges on which there were very few budding scars (Fig. 1). The presence of scars could affect the structure of the yeast cell wall. Data reported by Chaudhari et al. [16] suggest that the scars visualized with atomic force microscopy are much stiffer than other areas of the yeast cell wall. However, the scar occupies only approximately 1–2% of the cell surface, and this cannot be expected to have a significant impact on the higher stiffness and hardness of the cell. The emergence of a small number of scars after budding—and, thus, the inhibition of the proliferation process—was caused by stress conditions associated with the presence of selenium and the lack of nutrients in the culture environment (aqueous solutions).

The fraction of *C*. *utilis* and *S*. *cerevisiae* cells treated with selenium showed a deformation of the cytoplasmic wall and membrane. As a consequence of these processes, there was uneven thickening of the cell wall—a so-called folding. Some yeast cells reduced the pressure in the cell; therefore, the cell wall, which was not destroyed, became more wrinkled due to the formation of furrows. The information presented by Rabouille and Alberti [17] shows that, even in the absence of the cell wall, yeast cells that are under stress have a stiff structure and retain their shape.

**Fig. 2:**
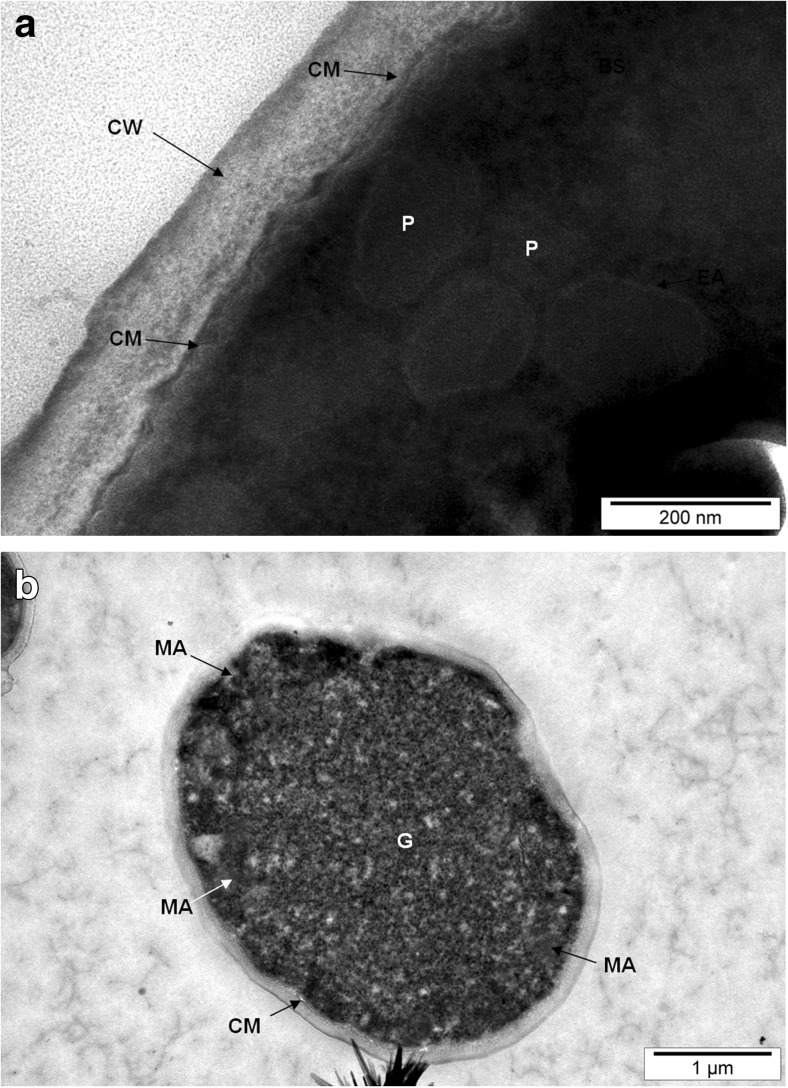
Image of *C*. *utilis* and yeast cells from 24-h culture in aqueous solutions enriched with 20 mg Se^4+^/L. **a** CM cell membrane, CW cell wall, P peroxisome. **b** G graininess, MA multimolecular assemblies

The *C*. *utilis* and *S*. *cerevisiae* yeast biomass obtained from aqueous solutions enriched with selenium were found to contain a multilayer cell membrane with a thickened inner layer (Fig. 2a). Therefore, the presence of selenium may reduce van der Waals forces in the lipid membrane, with resultant edema of the cell membrane. This edema negatively affects the energy barrier in the lipid layer of the membrane and changes the course of biochemical processes in the cells, thereby disrupting nutrient transport through the cell membrane [18]. In addition, we found irregularly shaped cells and concentrated cytoplasm. These effects may have been caused by either shrinkage or the occurrence of protoplast plasmolysis [19]. The cytoplasm of yeast involved in the accumulation of elements might become more acidic, which causes many proteins to bind to each other and form large clusters. As a consequence, the intracellular proton concentration is increased, which triggers the phase transformation of the cytoplasm from liquid to solid [20]. Moreover, yeast carries out the process of excess selenium detoxification to the red elemental form in its cellular structures. This process is enzymatic in nature and proceeds with the use of metabolic energy [2].

Dense granules were observed inside the yeast cells that were examined (Fig. 2b). Enclosing the yeast cells in an environment rich in selenium ions probably resulted in the formation of special chambers separated by a membrane as well as multimolecular complexes in the cellular cytosol. The resulting structures play a key role in the protection of cellular organelles [17]. The presence of selenium in the medium could affect the occurrence of many selenium–protein interactions, presumably through the remodeling of surface charges of these proteins, especially with a low isoelectric point; thus, this could lead to major changes in the organization and properties of the cytoplasm [21].

**Fig. 3:**
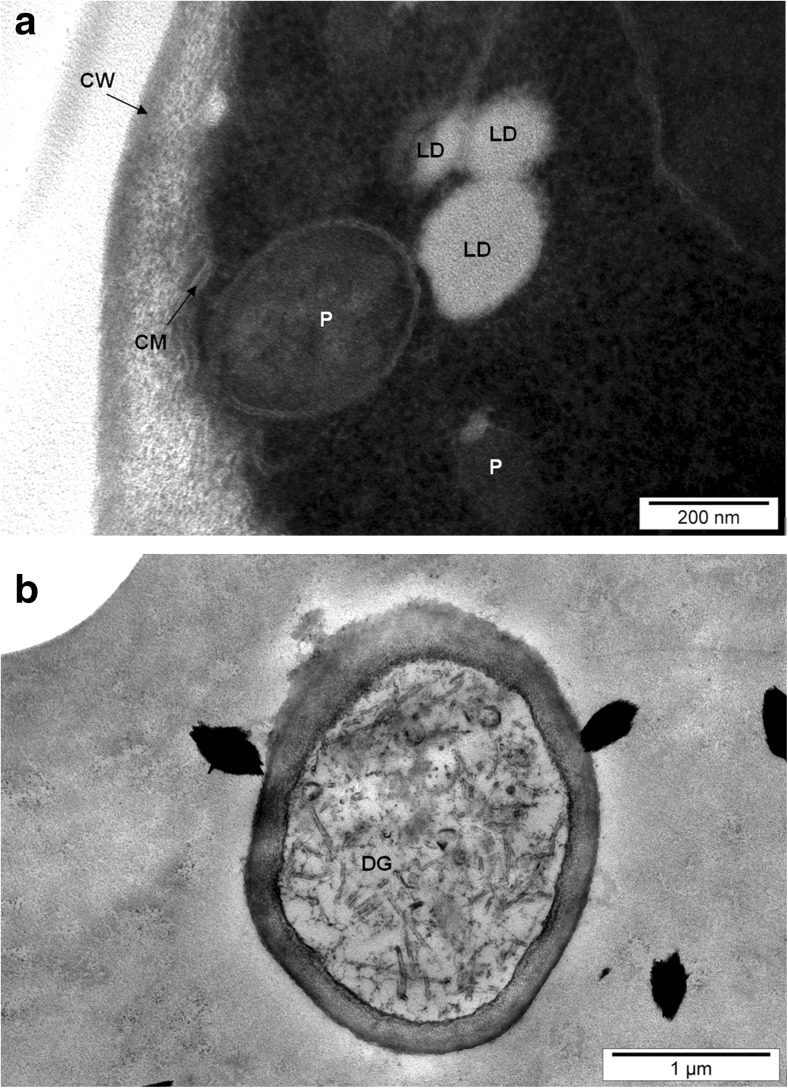
Image of *S*. *cerevisiae* yeast cells from 24-h culture in aqueous solutions enriched with 20 mg Se^4+^/L. **a** LD lipid drops, P peroxisome, CW cell wall, CM cell membrane. **b** DG degraded organelles

The presence of many lipid droplets could be observed in the yeast cytoplasm (Figs. 1 and 3a). The primary function of these lipid droplets is to participate in the storage and release of accumulated nutrients as a source of energy [22, 23]. In addition, they can be used as substrates for the synthesis of lipid components of cell membranes [24]. An effect of lipid droplets with various organelles is that they are treated as dynamic structures which play a key role in lipid metabolism and the maintenance of cellular energy homeostasis [25, 26].

**Fig. 4:**
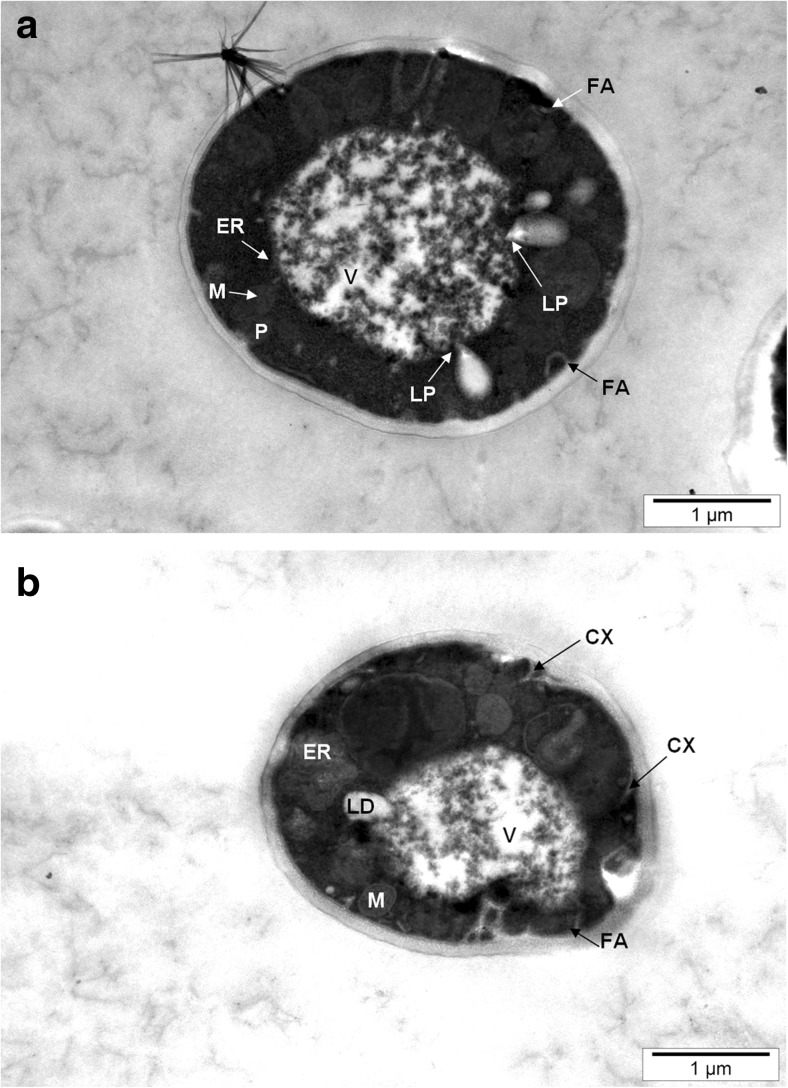
Image of *C*. *utilis* yeast cells from 24-h culture in aqueous solutions enriched with 20 mg Se^4+^/L. **a** ER endoplasmic reticulum, FA formation of autophagosomes, LP lipophagy process, M mitochondrial. **b** LD lipid drops, CX convex membrane

Typical vacuoles and other cell organelles could be easily identified in yeast cells obtained from aqueous solutions. Partially degraded cell organelles were visible in cross-sectional images of yeast cells cultured in the presence of selenium in the cytosol (Fig. 3b). Concurrent observations of two yeast species showed bubbles surrounded by a single protein–lipid membrane, called the autophagy body, inside the vacuole. The degradation of fat bodies in the lipophagic process was noted (Fig. 4a). To survive unfavorable culture conditions, the yeast cells used previously accumulated substances. Of note, under starvation conditions, dead or damaged elements of cell organelles could be used by the yeast in invagination processes involving a gradual deformation, which changes the curvature of the membrane to thereby cause it to be penetrated with the resultant formation of an autophagosome vesicle. This is a mechanism that is activated in response to stressful conditions and allows survival of yeast cells under adverse environmental conditions [27]. Furthermore, the progressive process of cell disorganization in the abovementioned conditions could lead to the release of autolytic enzymes, which cause the decomposition of intracellular organelles, but without the release of their contents to the extracellular environment. Such cells may be a source of food (nutrients) for the remaining yeast cells.

A small part of the selenium found in the yeast vacuole and cytosol can be moved extracellularly into the culture environment. Reports in the literature support the possibility of the occurrence of a number of mechanisms responsible for the removal of unsuitable substances from microorganisms. One of these is the phenomenon of outer membrane exaggeration, which arises in response to the stress associated with the presence of materials harmful to cells (Fig. 4b). Such toxic substances are moved to the extracellular compartment by lipid bilayer vesicles [19, 28]. In addition, transport proteins may participate in these processes [29]. Peroxisomes were probably observed in yeast cells (Figs. 2a, 3a, and 4a). This organelle plays an important role in the degradation of fatty acids in yeast cells; thus, *S*. *cerevisiae* and *C*. *utilis* become attractive organisms for research on this process. Peroxisomes contain enzymes that break down fatty acids and undertake cell detoxification [30].

Based on microscopic observations, we found that stress conditions due to the presence of selenium in aqueous solutions affected the disturbance of metabolic activity and structural stability of the examined yeast cells. However, the presented results do not fully elucidate the issue; they only indicate some information that would be helpful in further studies on the role of selenium in the metabolism of yeast cells. Therefore, it is important to conduct, in the future, further biochemical analyzes of selected elements affecting the transformation of selenium in yeast cells.

### Effect of Selenium on Protein Content

Yeast cell biomass can be used as an additive to feed as well as in the production of dietary supplements. This is evidenced by not only the very high protein content, but also the rich amino acid composition of this substrate [31, 32]. Yeasts that are characterized by high protein content and are attractive from a perspective of isndustrial production include *Candida*, *Hansenula*, *Pichia*, *Torulopsis*, and *Saccharomyces* [33]. Such a microbial protein is characterized by high digestibility that can approach 50%.

The examined biomass of *C*. *utilis* ATCC 9950 and *S*. *cerevisiae* MYA-2200 yeast obtained from aqueous solutions without added selenium was characterized by high total protein content that ranged from 42.1 to 48.4%. The increased concentration of this component in the yeast biomass was attributed to the presence of impurities in glycerol, because waste from the petrochemical industry may contain some amounts of peptides and proteins that are an additional source of nitrogen for yeast cells [34]. The second waste product used as an ingredient in the proliferation medium was potato wastewater—a rich source of protein compounds (2%), of which 78% constitutes soluble protein and non-protein nitrogen substances.

After cultivation in aqueous solutions supplemented with selenium, the protein content of both *S. cerevisiae* and *C. utilis* yeast strains was not significantly reduced in relation to the biomass obtained from solutions devoid of selenium, and was 42.6 and 37%, respectively (Table 1). The reduced protein content in yeast cells could be related to cellular adaptation to unfavorable environmental conditions and the expenditure of this nutrient on energy and metabolic processes. Cells must respond to changes in the availability of nutrients and the presence of additional microelements in the culture. Survival mechanisms involve inhibition of anabolic processes, such as protein synthesis in the cytosol [35]. As a consequence, the emerging stress conditions interfere with the functioning of yeast cells, thus affecting the occurrence of the denaturation process and the reduction of the mechanical force of the yeast cell wall [36].

**Table 1:** Protein content in yeast cell biomass

Yeast strains	The protein content in yeast biomass (%)	
	0 mg Se^4+^/L	20 mg Se^4+^/L
*C*. *utilis* ATCC 9950	42.1 ± 1.6^b^	37.0 ± 1.8^a^
*S*. *cerevisiae* MYA-2200	48.4 ± 1.2^b^	42.6 ± 1.3^a^

The advantage of the yeast biomass enriched with the appropriate dose of selenium ions is its nontoxicity and a huge potential for almost unlimited applications in food production [2, 37]. Its values are confirmed by not only the high protein content, but also its amino acid composition. Protein extracted from the yeast biomass can replace expensive and conventional (traditional) protein sources (e.g., soy) used in animal nutrition [33].

The protein contents in the *S*. *cerevisiae* and *C*. *utilis* yeast biomass recorded in this study were similar to those reported in the literature. The study conducted by Juszczyk and Rymowicz [38] showed that the protein content obtained from a *Yarrowia lipolytica* yeast biomass cultured in a medium containing crude glycerol was 42–45%. Results from a study presented by Dobrzański et al. [39] confirmed high protein content in the yeast enriched with selenium ions (35.8%). Moreover, the biomass of these microorganisms contained a higher content of leucine (2.81%), in comparison to the biomass without added selenium (1.25%). A slightly higher protein content in yeast biomass cultured on carbohydrate substrates was reported by Kurbanoglu [40]. In another study, the protein content obtained from the biomass of the *C*. *utilis* NRRL Y-900 strain cultured in a medium comprising wood hydrolysates ranged from 49.8 to 53%. The results of the study obtained by Stabnikova et al. [41] showed that protein content in *S*. *cerevisiae* yeast cultured in waste substrates from the fruit and vegetable industry ranged from 40 to 45%.

The decrease in the protein content in the biomass of both the examined yeast strains that were obtained from aqueous solutions enriched with selenium, compared to the control biomass devoid of selenium, could possibly be associated with the progressive process of lipid peroxidation and cell autolysis. Such results could consequently lead to the leakage of cytoplasmic material secondary to damage to the yeast–membrane complex of yeast cells [2].

In summary, it can be emphasized that optimization of deficit elements valuable in diet as well as protein from yeast micro-factories can be an alternative to proteins derived from plant or animal products. The production of microbial protein has a number of advantages. The most important include short time of microorganism generation, high content of suitable quality protein in the cells, and ability to shape the amino acid profile of proteins. Results from this study indicate that the selenium-enriched biomass of *C*. *utilis* and *S*. *cerevisiae* yeasts may be a valuable source of protein and selenium, which is deficient in the diet. This creates the possibility of obtaining selenium bioplexes that can be used in the production of protein–selenium preparations for human and animal applications.

### Effect of Selenium on Amino Acid Content in Yeast Biomass

Yeasts are used on a large scale as animal feed supplements. The popularity of these preparations as a source of protein increased at the beginning of the twenty-first century, when animal-origin meals were prohibited under certain conditions in animal feed in the European Union [39]. Yeast, in addition to containing valuable amino acids, is also a source of deficient elements. Amino acids are necessary for the growth and reproduction of cells, because they are the precursors for protein synthesis and participate in processes of nucleotide and lipid biosynthesis. Moreover, amino acids can be used as an energy “fuel” for all organisms.

**Fig. 5:**
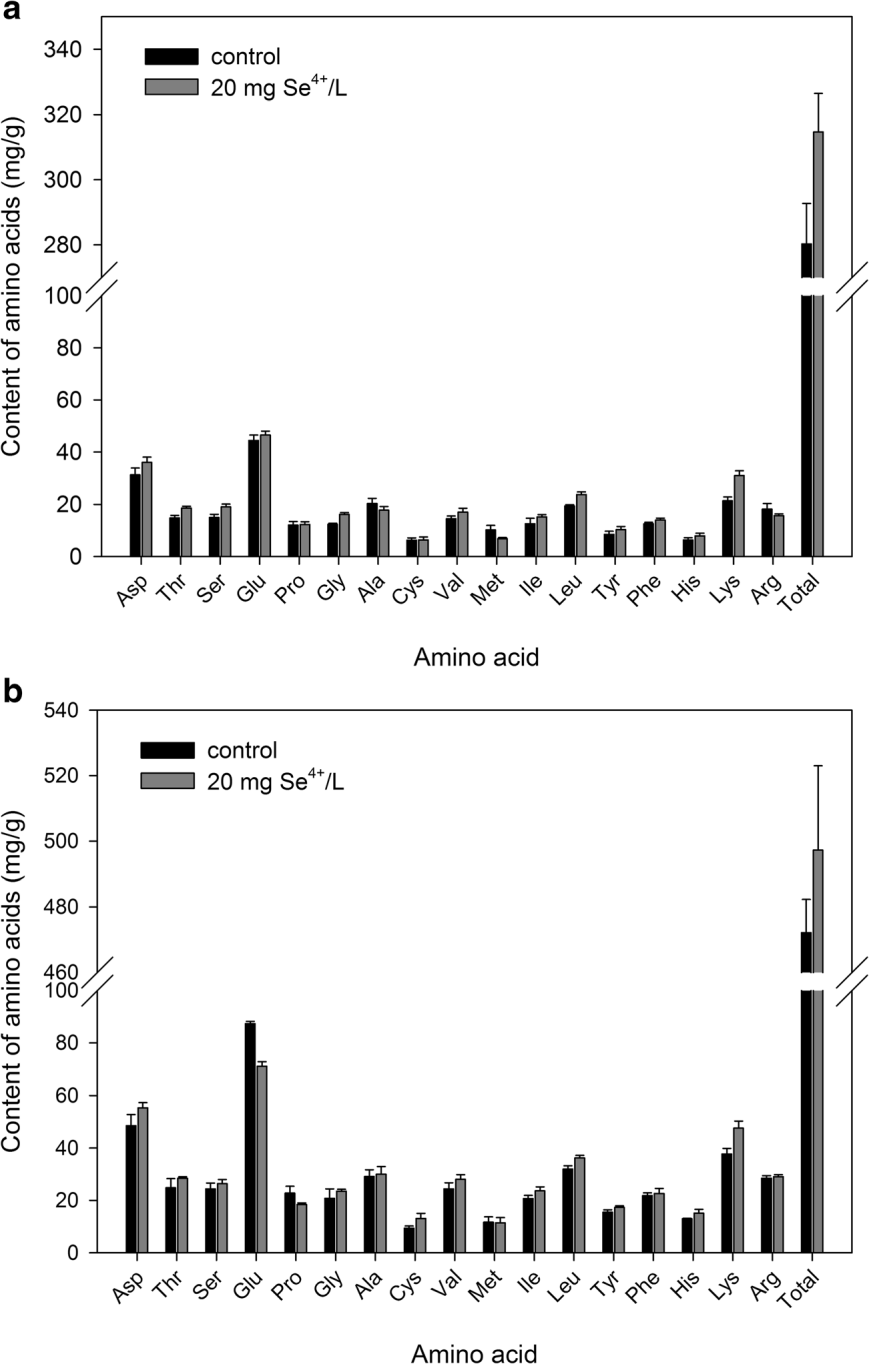
Amino acid content in *C*. *utilis* ATCC 9950 (**a**) and *S*. *cerevisiae* MYA-2200 (**b**) yeast biomass

Changes in protein content in the biomass of *C*. *utilis* and *S*. *cerevisiae* yeasts were accompanied by changes of amino acid composition. The average sum of all amino acids in the yeast biomass obtained from aqueous solutions without the addition of selenium was 280 and 472 mg/g_d.w._, respectively (Fig. 5a, b), of which the exogenous amino acids determining the nutritional value of protein accounted for approximately 46 and 45% of the total amino acid content, respectively. After culturing in aqueous solutions supplemented with selenium, the total amino acid content for both *C*. *utilis* and *S*. *cerevisiae* strains was higher, and increased by approximately 12 and 5%, respectively. The analysis of the content of some amino acids in the *C*. *utilis* biomass showed that yeast culture in aqueous solutions with selenium supplementation clearly decreased alanine content by approximately 2.56 mg/g_d.w._. Moreover, a slight decrease was observed for arginine (approximately 2.5 mg/g_d.w._) in relation to the biomass derived from the solution without selenium supplementation. In the case of *S*. *cerevisiae* yeast, there was no reduction in these amino acid contents. Attention to the levels of proline is necessary. Proline is an amino acid with protective functions in the cell that are manifested in the regulation of peroxidase and catalase enzymes [42]. So far, no detailed mechanism of action has been recognized for proline. The concentration of proline in the *C*. *utilis* biomass enriched with selenium, as well as in cultures without selenium supplementation, was comparable (approximately 12 mg/g_d.w._). In the case of *S*. *cerevisiae* yeast enriched with selenium, proline content decreased by approximately 4.3 mg/g_d.w._, as compared to that of the control biomass (Fig. 5b).

The *C*. *utilis* yeast biomass enriched with selenium was rich in glutamic acid (46.5 mg/g_d.w._), aspartic acid (36.0 mg/g_d.w._), and leucine (23.8 mg/g_d.w._) as compared to the control biomass. Slightly higher concentrations of amino acids—that is, glutamic acid (71.1 mg/g_d.w._), aspartic acid (55.3 mg/g_d.w._), leucine (36.2 mg/g_d.w._), threonine (28.5 mg/g_d.w._), and glycine (24.4 mg/g_d.w._)—were found in the *S*. *cerevisiae* biomass enriched with selenium. Suhajda et al. [43] found that protein content and concentration of individual amino acids in the yeast biomass depend on culture conditions and the presence of selenium in the culture environment.

The study presented by Wu et al. [44] showed that low methionine and high glutamic acid content can effect an increase in the viability of yeast cells. In addition, those authors demonstrated that the addition of glutamic acid to the culture medium with limited glucose content affected the prolongation of yeast viability. An increase in the concentration of this amino acid in *C*. *utilis* and *S*. *cerevisiae* yeasts enriched with selenium could be decisive in the adaptation of microorganisms to stressful environmental conditions.

The presence of selenium in aqueous solutions modulated changes in yeast cell metabolism. After it is accumulated by yeast, selenium can undergo reducing sulfur assimilation and transform into selenide, which is then used as a substrate for the synthesis of selenium amino acids, including SeMet [2, 45, 46].

Only amino acids determine the nutritional value of the protein. The most important of these are exogenous amino acids that are essential for humans and animals [44]. An analysis of the amino acid composition of *C*. *utilis* yeast biomass enriched with selenium showed that the concentration of the most important exogenous amino acids (lysine) was at a higher level (8.3%), as compared to that of the biomass obtained without the addition of this element (5.6%). In *S*. *cerevisiae* yeast enriched with selenium, the lysine content was approximately 10 mg/g_d.w._ higher as compared to that of the control sample (37.7 mg/g_d.w._). According to data in the literature, the concentration of lysine in cells of *C*. *utilis* yeast was approximately 7.1% [47]. Similar results were obtained by Michalik et al. [48] in a biomass of *Y*. *lipolytica* yeast cultured in glycerin media. The lysine content fluctuated around 6.2%. The concentration of lysine in the cells of *C*. *utilis* and *S*. *cerevisiae* yeasts in this study confirms the data reported by other authors.

The presence of mitochondria, which are energy-producing organelles, was observed in yeast cells (Figs. 1 and 4a, b). In addition, these organelles play a central role in the amino acid homeostasis. Under stress conditions resulting from the presence of selenium and the lack of carbon and nitrogen sources in the culture medium, mitochondrial metabolism of yeast cells is focused on the use of amino acids for energy needs. This leads to the operation of mechanisms aimed at increasing the levels of some amino acids in the yeast biomass. Thus, the lack of nutrients as well as the presence of selenium can induce an increase in the mitochondrial respiratory process resulting in the reinforcement of catabolic repression. As a consequence, the synthesis of cytosolic proteins is inhibited, which limits the yeast cell budding process.

The concentration of protein and amino acids in yeast can be mainly determined genetically; however, the skillful selection of appropriate ingredients and culture parameters may enable microorganisms to use their biotechnological potential. Bearing in mind the production of protein enriched with microelements (selenium) in the form of bioplexes with very good bioavailability and digestibility by human and animal organisms, such analyzes seem justified. Moreover, carefully planned acquisition of selenium-enriched yeast biomass will provide a reliable source of protein with the appropriate amino acid composition.

### Effect of Selenium on Fatty Acid Content in Yeast Biomass

Fatty acids are the basic units for the production of triacylglycerols (TAGs) in the esterification process, are the energy store for cells, and—together with phospholipids and glycolipids—they constitute the main components of cell membranes [49]. The total lipid content differed significantly among *C*. *utilis* ATCC 9950 and *S*. *cerevisiae* MYA-2200 yeast strains. In addition, significant differences in fatty acid profiles among the examined yeast strains enriched with selenium were noted in the present study.

**Fig. 6:**
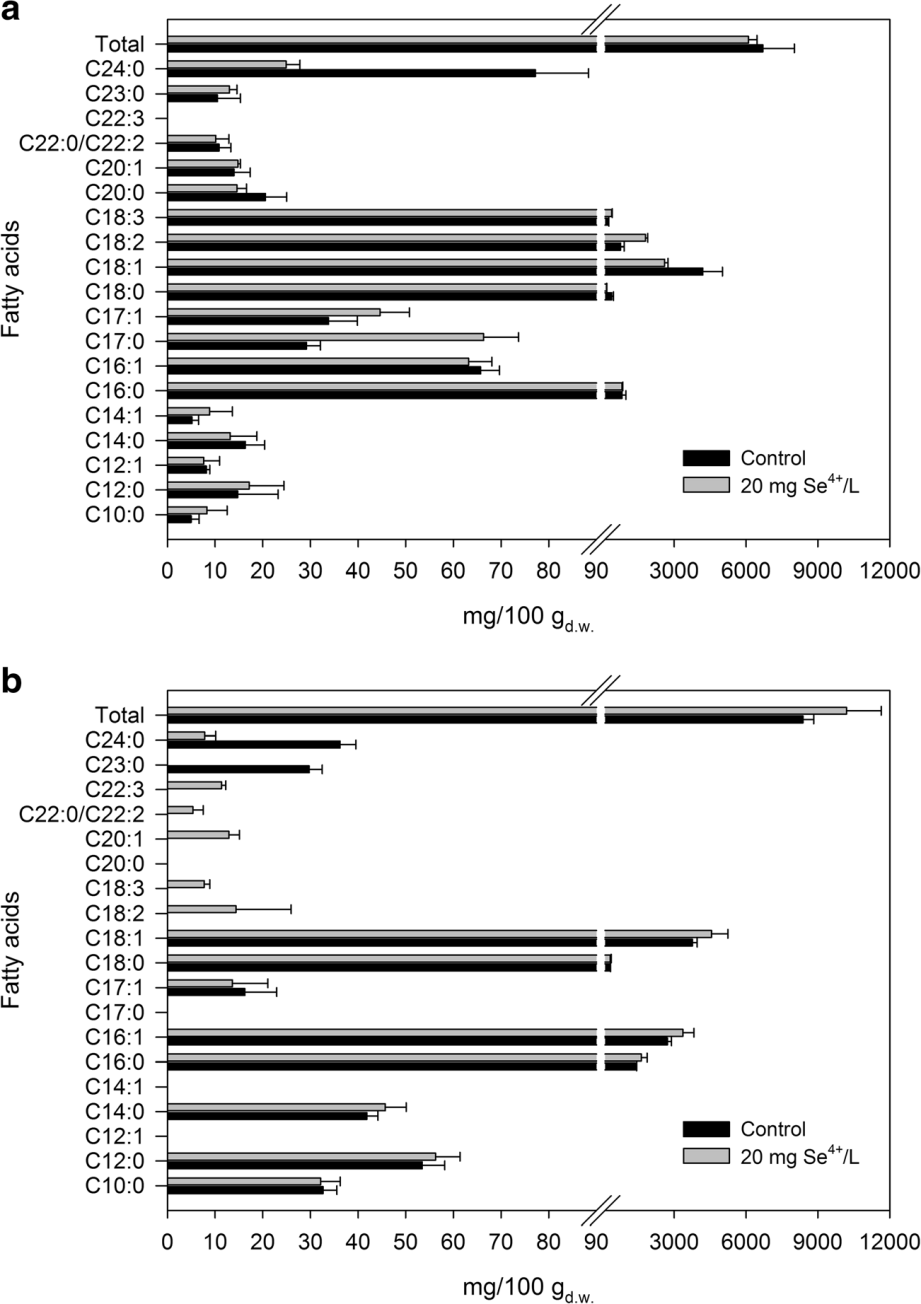
Fatty acid profile in *C*. *utilis* ATCC 9950 (**a**) and *S*. *cerevisiae* MYA-2200 (**b**) yeast biomass

The profile of fatty acids produced by yeast depends on the species, culture conditions, duration of culture, and temperature [13]. A very important parameter that affects lipid content is an appropriate carbon source [50]. The accumulation of lipids is triggered by the reduction of nutrients in the culture medium, in combination with carbon excess. To stimulate lipid accumulation, reduction of the nitrogen source is mainly used; however, other nutrients, such as phosphorus and sulfur, have been shown to induce lipid accumulation [51]. Oils and fats mainly consist of TAGs. Microorganisms use TAG as the basic form of carbon and energy source for metabolic needs [52]. Large droplets of TAGs can occupy the larger part of a cell. The main fatty acid found in yeast lipids is oleic acid (C18:1), and its share can be even higher than 70% of total fatty acid [14, 53]. The biomass of *C*. *utilis* yeast from the culture without added selenium was characterized by a significant amount of unsaturated fatty acids, including oleic acid (C18:1; 62%), linoleic acid (C18:2; 11%), and linolenic acid (18:3; 3%), in relation to the total fatty acid content (Fig. 6a). The results obtained are consistent with a report of the study presented by Chatzifragkou et al. [54], who noticed the advantage of C18 and C16 fatty acids, in particular oleic acid, in yeast cells. This fatty acid composition is similar to the composition of oil plants [54].

*C*. *utilis* biomass supplementation with selenium caused a reduction in the content of oleic acid by approximately 45% in relation to the total amount of fatty acids. In the case of the other two acids, their increase was to approximately 42 and 57% of the total fatty acid content. According to reports by Čertík et al. [14], yeasts cultured in a medium supplemented with selenium (1.2 mM) produced large amounts of linoleic acid and linolenic acid. Moreover, selenium in *Rhodotorula* yeast stimulates the biosynthesis of C18 fatty acids, as well as promotes the distribution of unsaturated C18 fatty acids in membrane lipids.

Palmitic (C16:0) and stearic (C18:0) acids are the major substrates of stearoyl-CoA1 desaturase (SCD1), and are converted to palmitoleic acid (C16:1) and oleic acid (C18:1), respectively [55]. This transformation resulted in a change in the length of the synthesized fatty acids in the biomasses of *C*. *utilis* and *S*. *cerevisiae* yeasts (Fig. 6a, b). In addition, the presence of free fatty acids (linoleic, stearic, and oleic acid) has a large impact on metabolite (including amino acids) synthesis in yeast cells (Samul et al. 2014). According to the data provided by Zhou et al. [56], an increased percentage of oleic acid (C18:1) and stearic acid (C18:0) in yeast can be attributed to the increased activity of a several enzyme complex called the fatty acid synthase (FAS). The yeast FAS system shows a much higher level of in vitro production of C16 fatty acids than do C18 fatty acids. Data in the literature [56] are consistent with the results obtained in our study. Moreover, a higher C16:0 content was found in the *S*. *cerevisiae* yeast biomass as compared to that of *C*. *utilis*. However, a higher palmitic acid content was only obtained in the *S*. *cerevisiae* biomass supplemented with selenium, as compared to that of the control biomass.

A high content of margaric acid (C17:0) and hexadecanoic acid (C17:1) was found in the biomass of *C*. *utilis* yeast enriched with selenium, at 66 and 44 mg/100g_d.w._, respectively. Furthermore, tetradecenoic (C: 14:1), linderic (C12:1), margaric (C17:0), and arachidonic acid (C:20) were found in the biomass of fodder *C*. *utilis* yeast, and these were not present in the biomass of *S*. *cerevisiae* yeast. These fatty acids—which are characterized by an activity beneficial to human and animal health including oleic acid (C18:1) with anticancer and anti-atherosclerotic properties or linoleic acid (C18:2) with a conjugated double-bond system (conjugated linoleic acid) of multidirectional health-promoting activity, being a precursor of arachidonic acid—can be distinguished in fatty acid profiles derived from the yeast cell biomass [57].

The presence of selenium in aqueous solutions led to an increase in the fatty acid desaturase activity [14], which consequently reduced the content of saturated fatty acids and increased the fluidity of yeast cell membranes [58]. Fatty acids are components of the cell membrane and are incorporated into the acyl chains and alkyl-1-ethyl chains of cellular lipids [18]. The modulation of the number and position of double bonds in acyl chains by individual fatty acid desaturases plays a key role in maintaining the appropriate dynamic state of the lipid bilayer [14, 57]. To develop properly, the yeast must meet two conditions. Firstly, there must be an adequate state of structure and firmness of the protein–lipid membrane that affects the turgor of the yeast cell. Secondly, the cytoplasm (density and composition of ions) must create a specific microenvironment, allowing stabilization of protein activity. The data reported in the literature data by Marova et al. [57] showed that the fatty acid composition of the yeast cytoplasmic membrane was modified in the presence of selenium within the culture medium. Čertík et al. [14], who investigated an effect of selenium on *Rhodotorula* and *Sporobolomyces* yeasts, found a change in the fatty acid profile of phospholipid fraction, which caused a decrease in the lipid bilayer fluidity affecting the viability of yeast, which consequently affected the metabolic activity of the cells.

The quantitative composition of fatty acids in yeast cells is subject to changes under the influence of various factors, including the presence of elements or the occurrence of stress conditions. In the case of the *S*. *cerevisiae* biomass enriched with selenium, the presence of linoleic, linolenic, eicosanoic, and docosahexaenoic acids was noted. A consequence of the formation of such long-chain forms was metabolic interconversion by enzymatic transformations of stearic and linoleic acid. Thus, the yeast cell can make a careful correction of the metabolic rate and, thereby, more efficiently maintain the balance between the energy demand and its acquisition. In the case of the biomass of *C*. *utilis* yeast enriched with selenium, the content of tricosylic acid (C23) increased by approximately 2.5 mg/100g_d.w._ in relation to biomass without this element, whereas the level of lignoceric acid (C24) decreased radically with respect to the biomass obtained without selenium supplementation. No tricosylic acid was found in the biomass of *S*. *cerevisiae* with selenium supplementation. In addition, lipids of *S*. *cerevisiae* yeast were characterized by a higher content of medium-chain fatty acids (C10–C14) compared to the biomass of *C*. *utilis* yeast. Such differences in fatty acid profiles can be used to identify new strains of microorganisms isolated from different environments [53].

Overall, the recognition of the mechanism responsible for changes in the fatty acid content in the yeast biomass in the presence of selenium is important from the practical perspective of their application in medicine and agriculture.

## Conclusion

This study provided preliminary information on the effect of selenium on the protein, amino acids, and fatty acid profile in the biomass of yeasts enriched with selenium. Selenium supplementation increased the share of unsaturated acids (e.g., linoleic acid, linolenic acid) in the biomass of *C*. *utilis* and *S*. *cerevisiae*. The biosynthesis of these acids may be associated with increased desaturase activity and lipid peroxidation. These processes affect changes in the morphology of yeast cells. The biomass of both strains enriched with selenium was found to contain a higher level of exogenous amino acids, including lysine, leucine, and valine, as compared to that of the control biomass. The results obtained in this study may be a starting point in research to explain the mechanisms of the selenium effect on amino acid and lipid metabolism processes in yeast cells. This would allow the conduct of further studies in the future aimed at understanding new biochemical pathways in which selenium participates. Nevertheless, yeast cells enriched with an appropriate dose of selenium that are characterized by a unique and beneficial amino acid composition certainly deserve further attention as a potential source of protein–selenium preparations. A new insight into these microorganisms could prompt scientists to look for ways to use the biochemical potential of yeast. New proposals for the use of these microorganisms and their metabolites in many industries will, furthermore, open up new practical and cognitive opportunities.
